# Golgi-associated retrograde protein (GARP) complex-dependent endosomes to trans Golgi network retrograde trafficking is controlled by Rab4b

**DOI:** 10.1186/s11658-024-00574-w

**Published:** 2024-04-16

**Authors:** Jérôme Gilleron, Abderrahman Chafik, Sandra Lacas-Gervais, Jean-François Tanti, Mireille Cormont

**Affiliations:** 1 Université Côte d’Azur, INSERM, Mediterranean Center of Molecular Medicine (C3M), Team “Insulin Resistance in Obesity and Type 2 Diabetes”, Bâtiment Archimed, 151 Route de Saint Antoine de Ginestière, BP 2 3194, 06200 Nice Cedex 03, France; 2 Université Côte d’Azur, CCMA, Centre Commun de Microscopie Appliquée (CCMA), Nice, France

**Keywords:** Endosome, *Trans* Golgi network, Cation-independent mannose-6-phosphate receptor, VPS52

## Abstract

**Background:**

The trafficking of cargoes from endosomes to the *trans*-Golgi network requires numerous sequential and coordinated steps. Cargoes are sorted into endosomal-derived carriers that are transported, tethered, and fused to the *trans*-Golgi network. The tethering step requires several complexes, including the Golgi-associated retrograde protein complex, whose localization at the *trans*-Golgi network is determined by the activity of small GTPases of the Arl and Rab family. However, how the Golgi-associated retrograde protein complex recognizes the endosome-derived carriers that will fuse with the *trans*-Golgi network is still unknown.

**Methods:**

We studied the retrograde trafficking to the *trans*-Golgi network by using fluorescent cargoes in cells overexpressing Rab4b or after Rab4b knocked-down by small interfering RNA in combination with the downregulation of subunits of the Golgi-associated retrograde protein complex. We used immunofluorescence and image processing (Super Resolution Radial Fluctuation and 3D reconstruction) as well as biochemical approaches to characterize the consequences of these interventions on cargo carriers trafficking.

**Results:**

We reported that the VPS52 subunit of the Golgi-associated retrograde protein complex is an effector of Rab4b. We found that overexpression of wild type or active Rab4b increased early endosomal to *trans*-Golgi network retrograde trafficking of the cation-independent mannose-6-phosphate receptor in a Golgi-associated retrograde protein complex-dependent manner. Conversely, overexpression of an inactive Rab4b or Rab4b knockdown attenuated this trafficking. In the absence of Rab4b, the internalized cation-independent mannose 6 phosphate receptor did not have access to VPS52-labeled structures that look like endosomal subdomains and/or endosome-derived carriers, and whose subcellular distribution is Rab4b-independent. Consequently, the cation-independent mannose-6-phosphate receptor was blocked in early endosomes and no longer had access to the *trans*-Golgi network.

**Conclusion:**

Our results support that Rab4b, by controlling the sorting of the cation-independent mannose-6-phosphate receptor towards VPS52 microdomains, confers a directional specificity for cargo carriers *en route* to the *trans*-Golgi network. Given the importance of the endocytic recycling in cell homeostasis, disruption of the Rab4b/Golgi-associated retrograde protein complex-dependent step could have serious consequences in pathologies.

**Supplementary Information:**

The online version contains supplementary material available at 10.1186/s11658-024-00574-w.

## Introduction

Endocytosis is a key cellular process required for communication between cells and their extracellular environment, which regulates cell growth and survival. Following endocytosis, cargoes (proteins, nutrients, and lipids) are delivered into early/sorting endosomes from which they are sorted to their intracellular destinations or recycled back to the plasma membrane [1, 2]. Defects in this pivotal cellular function have been implicated in human diseases, including neurological disorders, cancer, and metabolic diseases [2–4].

The trafficking of cargo from endosomes to the *trans*-Golgi network (TGN) is critical for a variety of physiological processes including lysosomal biogenesis, nutrient transport, or enzymatic processing of secretory polypeptides [5], for the pathogenic effect of some bacteria [6, 7], and can be dysfunctional in pathologies [8]. This retrograde transport is a specialized pathway used by a limited number of proteins, of which cation-independent mannose-6-phosphate receptor (CI-M6PR) is an archetype. Mechanistically, endosome-to-TGN retrograde transport is a multi-step process involving several complexes that act sequentially to allow the sorting of cargoes from endosomes and their arrival at the TGN. CI-M6PR is mainly sorted from endosomes to tubulovesicular carriers by interacting with the sortin nexin (SNX) - Bin, Amphyphysin, and Rvs (BAR;SNX-BAR) sorting complex for promoting exit-1 (ESCPE-1 complex), and to a lesser extent by the retromer [9, 10]. These endosome-derived carriers are then captured by the TGN by a long-distance tethering complex composed of the long coiled-coil Golgin proteins (GCC185, Golgin-245/p230, GCC88, and Golgin-97/GOLGA1) and a short-distance multisubunit tethering complex, the Golgi-associated retrograde protein (GARP) complex, which is composed of four subunits (VPS51, VPS52, VPS53, and VPS54) [11]. VPS54 is the subunit specific for the GARP complex, while the others, including VPS52, are shared with the endosome-associated recycling protein (EARP) complex. The GARP complex enables the assembly of the trans-SNARE complex, which is critical for the fusion with the TGN and thus for cargo delivery [12–15]. Small GTPases have been implicated in the localization of multisubunit tethering complexes (reviewed in [16, 17]). Recruitment of the Golgins and the GARP complexes at the TGN relies on Arl family of small GTPases, Arl1 and Arl5, respectively [15, 18]. For the long-distance tethering, TBC1D23 has been described to bridge the WASH complex on the endosome-derived vesicles to Golgin-245 or Golgin-97 at the trans-Golgi network [19, 20]. Their “amphipathic lipid-packing sensor” motif may also be sufficient to recognize the highly curved membrane of these vesicular carriers [21–23]. However, the small GTPase involved in the anchoring of the GARP complex to endosomes remains unknown.

To identify the small GTPase that links the GARP complex to endosome-derived vesicles, we searched for interactors of the GARP complex subunits in the BioGRID interaction repository database https://thebiogrid.org/. Several small GTPases, mainly of the Rab family, were found to be enriched near the different subunits of the GARP complex. Two of them, Rab4a and Rab4b were found to interact with VPS52 only in high-throughput screens [24, 25]. We did not consider Rab4a as a putative candidate because Rab4a co-localizes almost exclusively with the early endosomal marker EEA1 and because the Rab4a effector Rabip4/Rufy1 [26] is only very partially co-localized with Rab4b, while Rab4b decorates as yet unidentified subcellular organelles [27]. The aim of the present study was to investigate whether Rab4b is involved in the VPS52-dependent EE to TGN retrograde trafficking.

## Materials and methods

### Plasmids and siRNA

The constructions of pCDNA3-HA Rab4b, -HA-Rab4bQ67L, -S22N were described previously [27]**.** pCDNA3-myc-VPS52 was digested with BamH1 and Xho1. The purified VPS52 fragment was subcloned into BamH1/Xho1 predigested pACT2 (Clontech, TaKaRa, Kyoto, Japan), which allowed the conservation of the reading frame between the GAL4 activation domain and VPS52. Rab4bQ67L, -S22N, and N121I were subcloned into the pB27 plasmid. The sequences of the mutated forms of Rab4b were amplified by PCR using the Pfu polymerase and a forward primer surrounding the second codon of Rab4b with a flanking sequence for SpeI restriction enzyme and a reverse primer surrounding the stop codon with a flanking sequence for PacI restriction enzyme. The primers were designed to maintain the reading frame between the LexA DNA binding domain and Rab4b. The PCR products and pB27 were digested with SpeI and PacI, purified after migration on an agarose gel, and ligated. All the constructions were verified by DNA sequencing (GATC Biotech) and expression in the yeast strain L40.

The siRNA against Rab4b described in [27] was synthetized by Eurogentec (Seraing, Belgium). Si-RNA against VPS52 and VPS54, or control are ON-TARGET plus human siRNA-SMART pool L-011806-00 and 021174-00, respectively (Horizon Discovery, Cambridge, UK), the negative control being from Eurogentec or from Horizon Discovery. The siRNAs were used at a final concentration of 10 nM.

### Yeast two-hybrid

The yeast reporter strain L40 was co-transformed with the pB27 fusion proteins Rab4bQ67L, Rab4bN121I, and Rab4bS22N vector and pACT2-VPS52 using a lithium acetate base method and grown on a synthetic medium lacking leucine and tryptophan. Induction of the reporter gene LacZ was determined using the substrate X-Gal, as described in our previous work [26].

### Cell culture and transfection

Cells were grown at 37°C in an atmosphere containing 5% C0_2_. WT HeLa cells are the HeLa S3 cell line derived from cervical carcinoma cells. They were purchased from the American Type Culture Collection (Rockville, MD) in the 1980’s by Dr SJ O’Brien (Nova Southeastern University, Fort Lauderlade, Florida, US). They were obtained from Pr TL Cover (Vanderbilt University School of Medicine, Nashville, Tennessee, US). Cells were cultured in DMEM containing 5% fetal bovine serum and penicillin (100 U/mL) and streptomycin (100 µg/mL) (Invitrogen). HeLa cells stably expressing GFP-Rab4b as described in [27] were cultured as WT HeLa with the addition of G418 (0.4 mg/mL, Invitrogen). HeLa cells stably expressing GFP-GOLPH3 and CD8-CI-M6PR (a gift of M Seaman) were established by stable transfection of HeLaM cells provided by M.S. Robinson (University of Cambridge, Cambridge, UK) and were cultured as WT HeLa with the addition of G418 (0.4 mg/mL, Invitrogen) and puromycin (1µg/mL, Sigma), as described in [33]. The siRNAs were transfected using Oligofectamine^TM^ Transfection Reagent (Thermo Fisher Scientific) according to the manufacturer’s instructions. Plasmids were transfected using FuGENE®6 Transfection Reagent (Promega) with a ratio of 1:3 of plasmid/ FuGENE®6, according to the manufacturer’s instructions. Cells were tested for the presence of mycoplasma contamination once a month using a dedicated PCR test and by staining with DAPI.

### Internalization of transferrin, Cholera toxin, antibodies to CI-M6PR or CD8

One hour before ligand internalization, cells were serum starved in DMEM containing 5% bovine serum albumin (BSA). Fluorescent Tf (10 µg/mL), fluorescent Cholera Toxin (20 µg/ml), fluorescent anti-CI-M6PR, and anti-CD8 antibodies were incubated with cells in DMEM/5 % BSA, as described in the legends of the figures, before proceeding for immunofluorescence. Anti-CD8 was detected with anti-species secondary antibodies (Table 1).

**Table 1:** List of antibodies and ligands

Protein	Reference	Application
CD8	Mouse monoclonal UCHT4, BIOMOL GmbH #153-020	Binding/IF
CI-M6PR	Mouse monoclonal MEM-238, EXBIO # 11-315-C100	IF/TEM/WB (non-reduced)
EEA1	Rabbit monoclonal C45B10, CST #3228	IF
	Mouse monoclanal, BD Biosciences #610456	IF
GFP	Mouse monoclonal 270F3, Synaptic Systems #132 011	IF/TEM
	Polyclonal BD Living colors, BD Biosciences # 632460	IF/TEM
GM130	Mouse monoclonal, BD Biosciences #610822	IF
HA	Rabbit polyclonal, MBL #561	IF
	Mouse monoclonal HA.11, Covance #MMS-101P	IF/WB
c-Myc	Mouse monoclonal 9E10, Biolegend #62,801	WB
p230	Mouse monoclonal, BD Biosciences #611280	IF
Rab4b	Rabbit polyclonal anti mouse C-terminal Rab4b [40]	WB
Sortilin/NT3	mMuse monoclonal, BD Biosciences #612100	WB
TGN46/TGOLN2	Rabbit polyclonal, AbD serotec #AHP1586	IF/TEM
Alpha-tubulin	Mouse monoclonal, Sigma #56199	WB
VPS52	Rabbit poyclonal #HPA042667 Atlas Antibodies or gift [47]	WB
	Mouse polyclonal, Abnova #H00006293-B01	IF

ExBio, MBL (Clinisciences SAS, Nanterre, France); Cell Signaling technology, Atlas Antibodies (Ozyme Saint-Cyr-L’Ecole, France), BD Biosciences (Beckton Dickinson, Le Pont-de Claix, France), Synaptic Systems, Abnova (Interchim, Monluçon, France), Covance Antibody (Eurogentec, Seraing, Belgium), AbD Serotec (Bio-Rad, Marnes-La Coquette, France), Sigma-Aldrich (Saint Quentin Fallavier, France),, Biolegend Europe BV (Amsterdam, the Netherlands)

Alexa Fluor™-coupled anti-species and anti CI-M6PR (MEM) antibodies, cholera toxin, and transferrin are from Invitrogen (Fisher Scientific, IllKirch, France). DyLight™-coupled and HRP-coupled anti-species antibodies are from Jackson ImmunoResearch Laboratories (Cambridgeshire, UK). Gold-coupled anti species antibodies were from BBInternational (Cardiff, UK)

### Transmission electron microscopy (TEM), immunofluorescence, confocal imaging, and image analyses

Immunolabeling and TEM were performed at the GIS-IBiSA-labeled “Microscopie Imagerie Cytométrie Azur” MICA platform of the “Centre Commun de Microscopie Appliquée” (CCMA) as previously described in [27].

Cells were grown on glass coverslips for immunofluorescence. At the end of the internalization assay, cells were washed twice in ice-cold PBS, fixed in 4% paraformaldehyde for 15 min, washed twice in PBS, incubated in 10 mM NH_4_Cl, and washed twice in PBS. They were then permeabilized with PBS containing 0.1% Triton X-100, 1% BSA, and 1% SVF for 30 min. They were then incubated with primary antibodies prepared in PBS containing 1% BSA and 1% SVF for 1h at room temperature, washed once in permeabilization buffer and twice in PBS/BSA/SVF, and then incubated with the appropriate fluorescent anti-species antibodies for 1h at room temperature. After washing, coverslips were mounted on slides using Mowiol 4-88 in a glycerol-based mounting medium. Images were acquired using confocal microscopes available on the platform IBiSA MICA of the C3M (either a LSM510 META confocal microscope with a Carl Zeiss SAS PL APO 63x 1.40 NA oil immersion objective or an A1R confocal microscope with a CFI Plan Apo Lambda 60 x 1.40 NA oil immersion objective from Nikon Instruments. Confocal parameters (pinhole, laser intensity, detector gain) were fixed within an experiment. Image processing and quantification were performed using ImageJ software. For fluorescence intensity quantification, the same threshold was applied to all images. Co-localization indexes were determined using the JACoP plugin or Imaris (OXFORD Instruments). For object-based quantification we used Imaris 3D-series of confocal images and then we applied a 3D-surface rendering for GFP-Rab4b, p230, or internalized florescent antibodies to CI-M6PR. The VPS52 signal on structures smaller than 150 nm was converted to objects. We then selected VPS52 dots close (less than 0.150 µm) to GFP-Rab4b, p230 surface, or iCI-M6PR rendering volumes. For Super-Resolution Radial Fluctuation (SRRF), high-speed time-lapse of 200 images were acquired at full speed on the NIKON A1R confocal microscope using the resonant scanner, to obtain fluctuation of the fluorescence signals. We then applied the NanoJ-SRRF plugin (ImageJ) to obtain super-resolution images and we realigned the different channels based on the chromatic shift estimated from TetraSpeck beads (ThermoFisher T7279).

### Western Blot, centrifugation on density gradient, and RT-qPCR

Western blots were performed as described in [27]. RT-qPCR was performed using standard protocols with RPLP0 expression for normalization. Primers for human Rab4b: Forward tcc caa gag ctc cca aag c; Reverse cca ggt aat aaa taa cag gta cta gca aca. Primers for RPLP0: Forward gc atc agt acc cca ttc tat cat; reverse ag gtg taa tcc gtc tcc aca ga.

### Statistical analyses

Statistical analyses were performed using the GraphPrism software (GraphPad from Dotmatics). For statistical significance between two groups, we first assessed whether the distribution was symmetric or asymmetric using the Shapiro-Wilk normality test. For normal distribution, we applied the Student T-test without the Welch correction for homogeneous variance, or with Welch correction if the variance was found to be heterogeneous using the F-test. For asymmetric distribution, the Mann & Whitney test was used. For statistical significance between more than two groups, one-way ANOVA was used. Irrespective of the test used, a difference was considered statistically significant if the p-value was < 0.05. All statistical details of the experiments are given in the figure legends.

## Results

### Rab4b is shuttling between Early Endosome Antigen 1 (EEA1)-positive endosomes and the *trans*-Golgi network

**Fig. 1:**
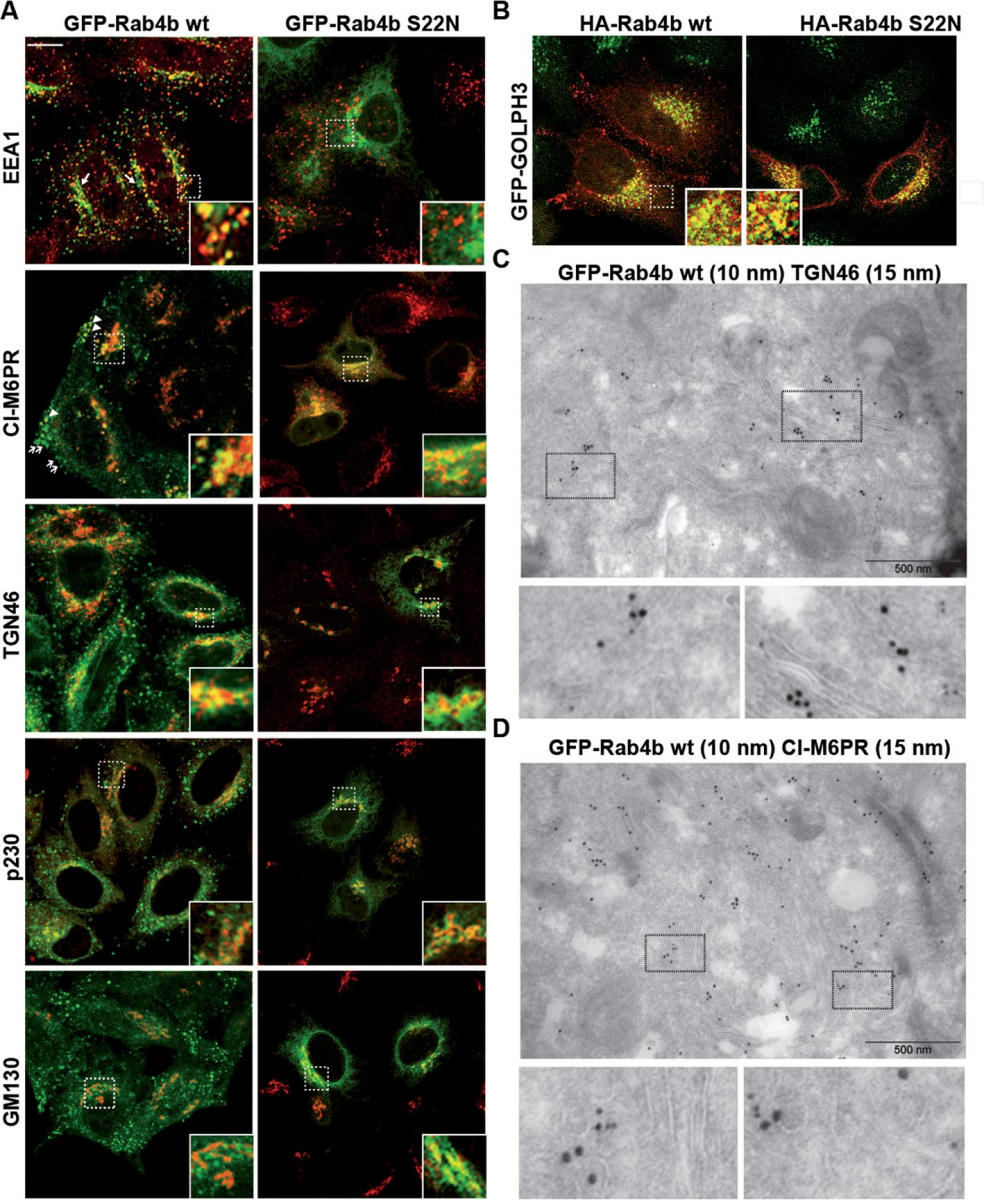
Localization of Rab4b wt and S22N. **A** EEA1, CI-M6PR, TGN46, p230, or GM130 labeling (red) in HeLa cells expressing the same amount of GFP-Rab4b (wt) or GFP-Rab4b S22N (green) shown as merged images. were treated for immunofluorescence using antibodies and anti-species Texas Red-coupled secondary antibodies. The bar corresponds to 10 µm. Enlarged views of the demarcated area are also shown. Arrowheads indicate to GFP-Rab4b-positive vesicles containing M6PR, whereas arrows point to Rab4b-positive structures not containing M6PR. **B** merged images of GFP-GOLPH (green) and HA-tag (red) labeling. The bar corresponds to 10 µm. Enlarged views of the delineated area are shown. **C**, **D** TEM images of GFP-Rab4b (10 nm gold particles) and TGN46 **C** or CI-M6PR **D** (15 nm gold particles) using mouse anti-GFP and rabbit anti-TGN46 **C** or rabbit anti-GFP and mouse anti-CI-M6PR (**D**). Enlarged views of the delineated area are shown and bars are for 500 nm

**Fig. 2:**
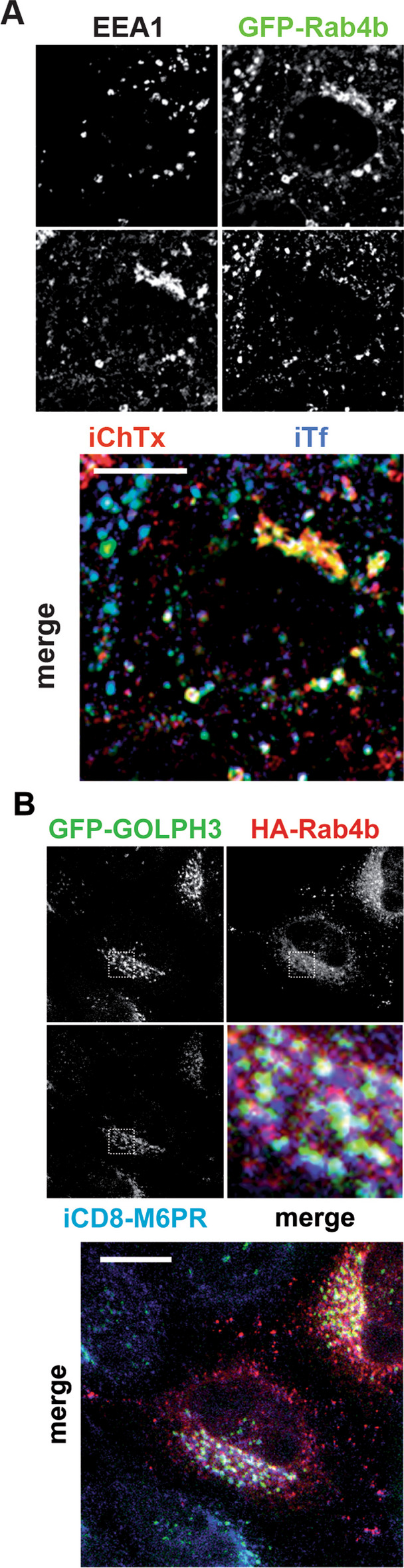
Cargoes accessibility. **A** Labeling of co-internalized Tf (iTf) and ChTx (iChTx), and of EEA1 in GFP-Rab4b expressing cells shown individually in white and black (left). The merged image of iTf (blue), iChTx (red), and GFP-Rab4b (green) (right). **B** Labeling of internalized CD8-M6PR (iCD8-M6PR, blue), in cells expressing GFP-GOLPH3 (green) and HA-Rab4b wt (red) shown individually in black and white, or as a merged image. Bars are for 10 µm

To study whether Rab4b was involved in early endosome to TGN retrograde trafficking, we first better characterized its subcellular localization. We characterized the distribution of Rab4b between intracellular organelles in HeLa cells stably expressing low amounts of Green-fluorescent protein-Rab4b (GFP-Rab4b), in order to minimize changes in organelle morphology that could be caused by a strong overexpression [27]. GFP-Rab4b was found colocalized with early endosomes (EEA1), the TGN (p230), and to a lower extent with the cis Golgi (GM130). Whereas nearly all the EEA1-positive endosomes were decorated by GFP-Rab4b, around 60% of GFP-Rab4b-positive structures were devoid of EEA1 (Additional file 1: Fig. S1B). The distribution of these GFP-Rab4b-positive and EEA1 negative compartments appeared bimodal with some structures at the cell periphery and some others in the perinuclear region. Because we previously characterized the peripheral vesicles as endosomes accessible to internalized transferrin (Tf) [27], we specifically studied the uncharacterized GFP-Rab4b-positive perinuclear tubular compartment (Fig. 1A, arrows in the panel showing EEA1 and GFP-Rab4b wt co-labeling). In these compartments, Rab4b was partly co-localized with CI-M6PR (cation-independent mannose-6-phosphate receptor) and TGN46 (also named *trans* Golgi network integral membrane protein 2, TGOLN2), two cargoes known to be recycled from the endo-lysosomes back to the *trans* Golgi network (TGN) (Fig. 1A). Consistently, ultrastructural exploration of GFP-Rab4b positive compartments by immuno-gold electron microscopy revealed that Rab4b was associated with tubulo-vesicular structures near the Golgi stacks and co-labeled with TGN46 (Fig. 1C) or CI-M6PR (Fig. 1D). In addition, we found that these compartments were partly co-labeled with the endogenous *trans* Golgi coiled-coil protein p230/golgin-245 (Fig. 1A) and over-expressed GOLPH3 (Fig.1B), but were only weakly co-labeled with the *cis* Golgi marker GM130/GOLGA2 (Fig. 1A and for quantification Additional file 1: Fig. S1A). To functionally discriminate between early endosomes and the TGN, which are very close in the perinuclear region of HeLa cells, we used two fluorescently labeled cargoes, the B subunits of cholera toxin (ChTx) and Tf, which, when internalized at steady state, labeled either the endosome to TGN retrograde pathway or the early and recycling endosomes, respectively (Fig. 2A). The perinuclear compartments labeled by GFP-Rab4b that accumulated the ChTx but not Tf (in yellow) probably corresponded to the TGN. Using the HeLa cell line stably expressing GFP-GOLPH3, which is located at the *trans* Golgi, and the chimeric retrograde cargoes consisting of the CI-M6PR fused to an extracellularly exposed CD8 antigen, we showed that internalized anti-CD8 antibodies trafficked back to perinuclear structures positive for both HA-Rab4b and GFP-GOLPH3 (white compartments in the image with the merge of all channels, Fig. 2B). Altogether, our results highlight that Rab4b is decorating the TGN in addition to early endosomes.

It was described that Rab GTPases cycle between an active GTP-bound form and an inactive GDP-bound form to coordinate membrane trafficking between two adjacent intracellular compartments. According to the current knowledge, inactive Rab GTPases are first recruited to the donor compartment and activated by the guanine nucleotide exchange factor (GEF). Afterwards, active Rab GTPases drive, through the recruitment of its effectors, the trafficking steps toward the target compartment, before its inactivation by the GTPase activating protein (GAP) [28, 29]. Therefore, we characterized where Rab4b-S22N, an inactive Rab4b protein accumulated. We found that Rab4b S22N was not localized in EEA1-positive early endosome (Fig. 1A and Additional file 1: Fig. S1A-B), but was detected in the TGN, where it colocalized with CI-M6PR, TGN46, p230, and GOLPH3 (Fig. 1A, B and Additional file 1: Fig. S1A). Moreover, a colocalization with the *cis* Golgi marker GM130 was also found (Fig. 1A and Additional file 1: Fig. S1A). These results suggest that GDP-bound Rab4b is associated with the Golgi, whereas GTP-bound Rab4b is rather present in early endosomes and peripheral vesicular structures. Hence, it is likely that Rab4b controls a trafficking step between endosomes and the TGN.

### Retrograde transport to the *trans-*Golgi network is dependent on Rab4b

**Fig. 3:**
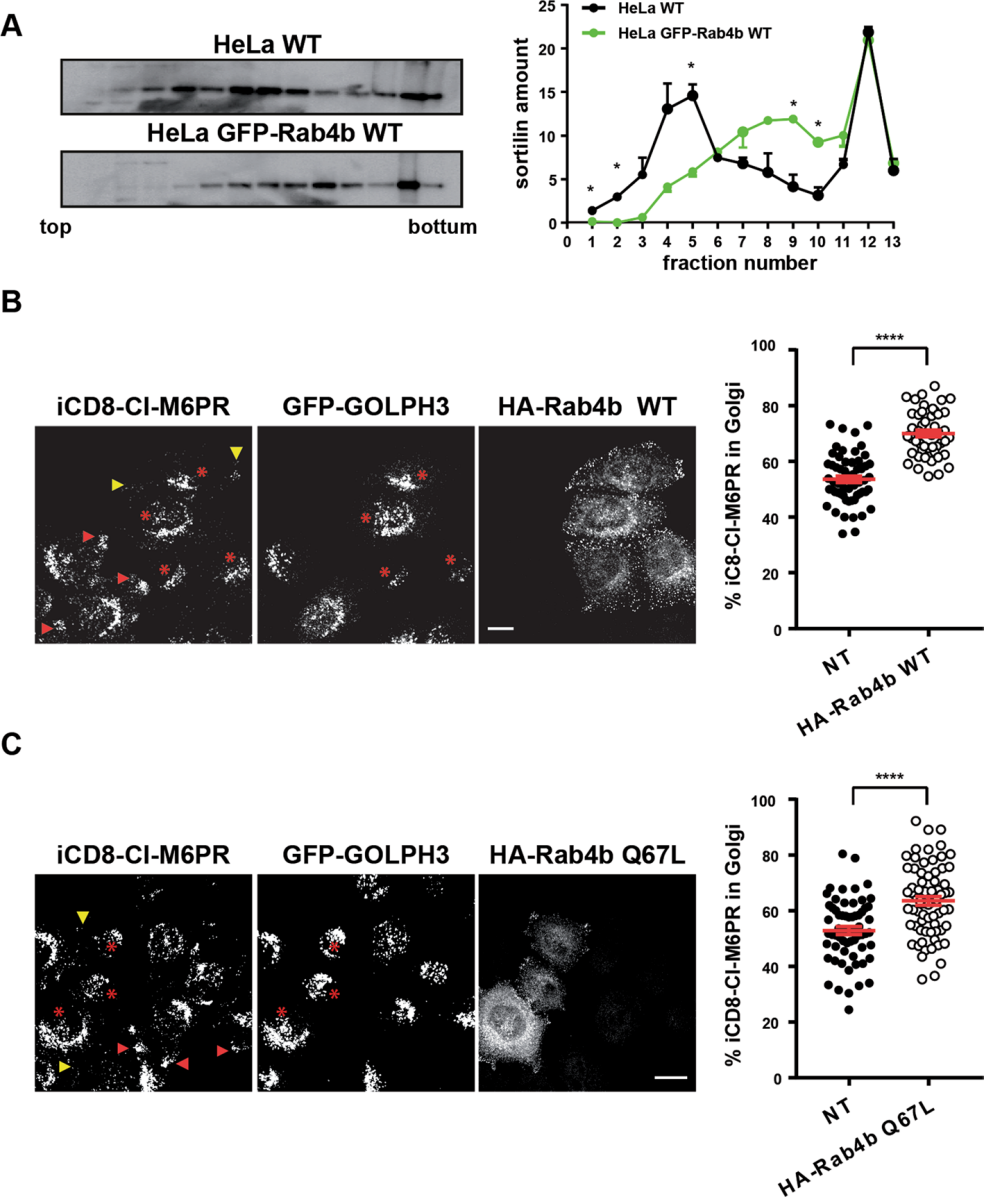
Active Rab4b favors retrograde trafficking to TGN. **A** Western blot of sortilin on homogenates separated on a glycerol gradient prepared from HeLa cells or HeLa cells expressing GFP-Rab4b wt. Representative blots and the quantification of 4 independent experiments. * indicate that the differences are significant with p < 0.05 (One Way ANOVA) **B**, **C** Quantification of the percentage of iCD8-M6PR in the Golgi area labelled with GFP-GOLPH3 in single cells expressing HA-Rab4b wt **B** or HA-Rab4b Q67L **C** compared to the surrounding and non-transfected cells. Representative images are shown (left) using the protocol 1 described in Additional file 1: Fig. S2A. Red stars indicate the HA-Rab4b expressing cells. Red and yellow arrowheads point to the periphery of the cells in cells expressing or not-expressing Rab4b, respectively. *** indicates that the differences are significant with p < 0.0001 (student t test). The bars are for 10 µm

We next determined whether Rab4b was indeed involved in the retrograde transport to the TGN. First, we determined the consequences of the over-expression of Rab4b on the subcellular localization of Sortilin and/or CI-M6PR. These two proteins act as carriers for lysosomal enzymes, loading them at the level of the TGN. They then traffic from the TGN to the plasma membrane and/or the early endosomes, where they release the lysosomal enzymes into the endosome lumen, allowing them to reach late endosomes and lysosomes. The carriers are recycled from the endosomes back to the TGN by the retrograde trafficking pathway (for review [30]). Using density centrifugation on glycerol gradients, we observed that the overexpression of wt GFP-Rab4b increased the density of the intracellular compartments containing Sortilin (Fig. 3A).

**Fig. 4:**
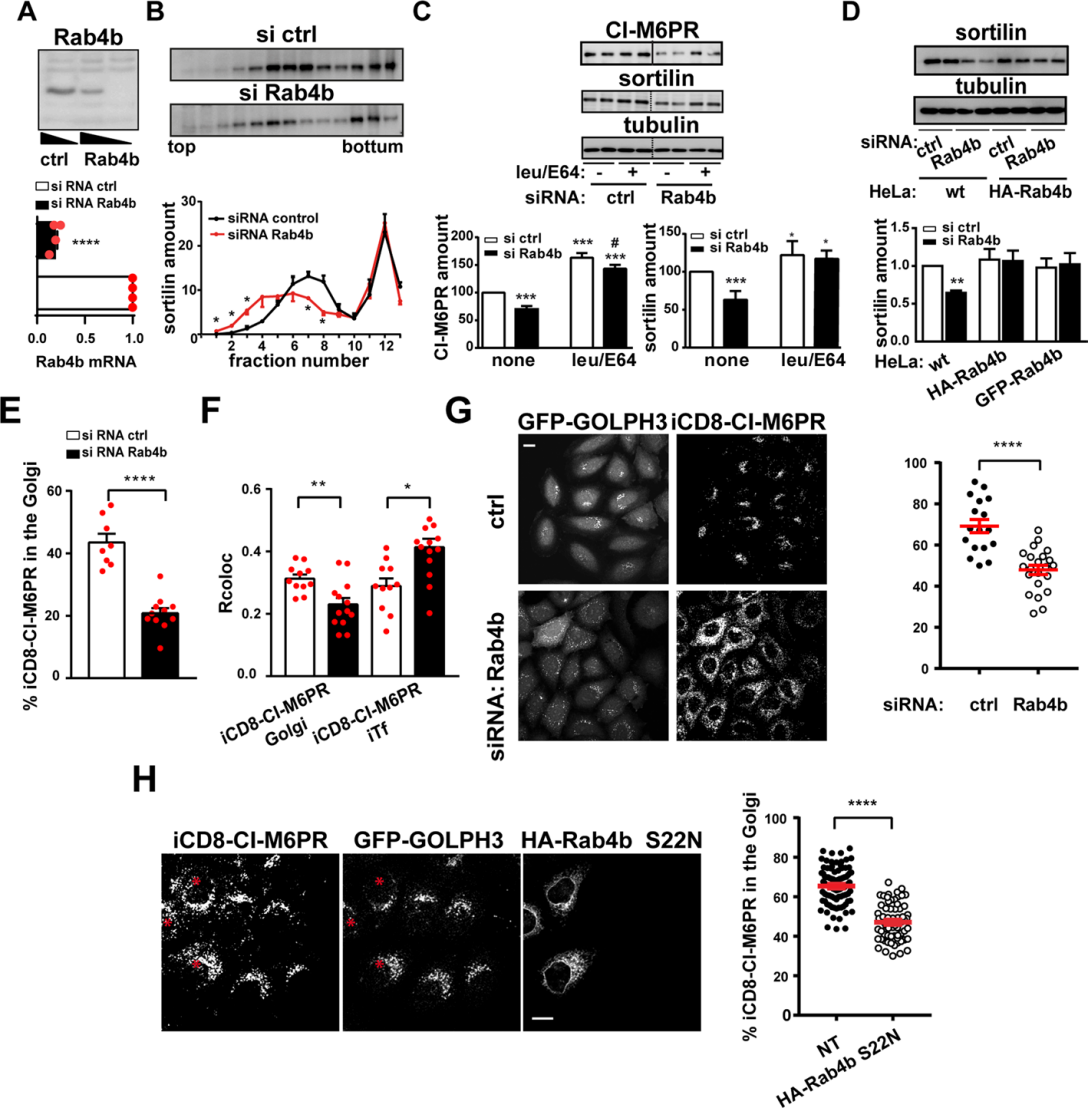
Inactive Rab4b blocks retrograde trafficking to the TGN. **A** Western blots of Rab4b on lysates prepared from cells transfected with (ctrl: 30 nM) or anti-Rab4b siRNA (5, 30 nM) as described in [27]. Quantification of Rab4b mRNA expression in cells treated with 20 nM ctrl or anti-Rab4b siRNA. **B** Western blot of sortilin as in Fig. 3A from control- or Rab4b siRNA-treated cells, and quantification of 3 experiments. *, p < 0.05 (two-way ANOVA). (**C**) Western blots of CI-M6PR, sortilin, and tubulin as loading control from controlor Rab4b siRNA-treated cells incubated without or with leupeptin and E64 and the quantification of 3 independent experiments. Significance compared to the ctrl/no leu/E64 condition with * p < 0.05, ** p < 0.01, *** p < 0.001; significance compared with the Ctrl within the condition no leu/E64 and leu/E64 ^#^ p < 0.05 (one-way ANOVA). **D** Western blots of sortilin, and tubulin as loading control, from control or Rab4b siRNA treated wt cells or Rab4b overexpressing cells. **E** Quantification of the percentage of iCD8-M6PR at the Golgi in randomly acquired whole fields in control- or Rab4b siRNA-treated cells using the protocol 1 (Additional file 1: Fig. S2A). **F** I colocalization index (RColoc) between iCD8-CI-M6PR and Golgi, or iCD8-CI-M6PR and co-internalized Tf, determined in the same experiments as in E, using the colocalization plugin of the open ImageJ software [51]. **G** Quantification the percentage of iCD8-M6PR at the Golgi in randomly acquired whole fields in control- or Rab4b siRNA-treated cells using the protocol 2 (Additional file 1: Fig. S2A) and representative images on the left panel. H Quantification of iCD8-M6PR in the Golgi of single cells overexpressing HA-Rab4b S22N (red star marks) and in the surrounding non-transfected cells (NT) and representative images according to protocol 1 (Additional file 1: Fig. S2A). **E–H** Significance compared to siRNA ctrl condition with *p < 0.05, **p < 0.01, ****p < 0.0001 (Student t test). Bars are for 10 µm

We then investigated the effect of the overexpression of HA-Rab4b WT or a constitutively active form of Rab4b (HA-Rab4b Q67L) on the retrograde trafficking of CI-M6PR to the Golgi. For that, HeLa cells stably expressing CD8-CI-M6PR and GFP-GOLPH3 were transiently transfected to express HA-Rab4b WT or HA-Rab4b Q67L and then incubated with anti-CD8 antibodies at 37°C for 1h. In non-transfected cells (NT), the overexpressed CD8-CI-M6PR fusion protein that is internalized (hereafter referred to as internalized CD8-CI-M6PR, abbreviated iCD8-CI-M6PR) was found both in the Golgi and in the periphery of the cells (red arrowhead). iCD8-CI-M6PR localized in the periphery of the cells most likely labelled early endocytic compartments because they disappeared when a pulse-chase experiment was performed (see Fig. 4H and Additional file 1: Fig. S2C). In cells transfected with HA-Rab4b WT (Fig. 3B) or HA-Rab4b Q67L (Fig. 3C), the targeting of iCD8-CI-M6PR to the Golgi was more efficient. Only a faint signal of anti-CD8 antibodies was observed in HA-Rab4bQ67L-positive and GFP-GOLPH3 negative structures at the edge of the cells (yellow arrowhead). Thus, Rab4b overexpression favors CD8-CI-M6PR retrograde trafficking to the TGN and its GTPase activity is not required for this trafficking pathway.

We next determined whether Rab4b was necessary for endosomes to TGN retrograde trafficking. First, we knocked-down the expression of Rab4b by RNAi gene silencing as in [27] (Fig. 4A) and studied the consequences on Sortilin and/or CI-M6PR (Fig. 4C–G). Using density centrifugation on glycerol gradients, we observed that, in contrast to GFP-Rab4b WT overexpression (Fig. 3A), the Rab4b silencing reduced the density of the intracellular compartments containing Sortilin (Fig. 4B). Furthermore, the protein levels of CI-M6PR and Sortilin were also decreased (Fig. 4C, D). This decrease in expression was prevented by inhibition of lysosomal proteases with leupeptin and E64 (Fig. 4C), indicating that in the absence of Rab4b, Sortilin and CI-M6PR are misdirected to the lysosomes where they are degraded. Importantly, Sortilin expression was rescued in cells expressing a form of Rab4b insensitive to Rab4b siRNA (Fig. 4D). In addition, we knocked down Rab4b in HeLa cells stably expressing CD8-CI-M6PR and GFP-GOLPH3 and we determined the percentage of internalized anti-CD8 antibodies bound to CD8-CI-M6PR that reach the Golgi at steady state (Additional file 1: Fig. S2A condition 1). We found that this percentage is decreased by 2-fold while the index of colocalization with concomitantly internalized fluorescent Tf is increased (Fig. 4E–G and Additional file 1: Fig. S2B for representative images). Similar observations were made for the ChTx (Additional file 1: Fig. S2C). To determine whether Rab4b altered CD8-CI-M6PR sorting at the early endosomes, we performed a functional early endosome to TGN retrograde assay in the HeLa cells, depleted or not for Rab4b, and stably expressing CD8-CI-M6PR and GFP-GOLPH3. To do so, we loaded the early endosomes with internalized CD8-CI-M6PR by incubating the cells with anti-CD8 antibodies for 15 min at 20°C [31]**,** before unleashing its trafficking by shifting to 37°C for the next 30 min (Additional file 1: Fig. S2A condition 2). We observed that the downregulation of Rab4b expression decreased the percentage of internalized CD8-CI-M6PR that reached the Golgi apparatus (Fig. 4G), as did the downregulation of VPS54 which is specifically involved in this retrograde trafficking pathway [32] (Additional file 1: Fig. S2D). Because Rab4b is required but its GTPase activity appears to be dispensable for its function in the retrograde trafficking pathway (see Fig. 3C), we determined whether GTP binding is needed. We expressed a form of Rab4b unable to bind GTP (HA-Rab4b S22N) in HeLa cells stably expressing CD8-CI-M6PR and GFP-GOLPH3 and incubated them for 1h at 37°C with anti-CD8 antibodies. In cells overexpressing HA-Rab4b S22N, the percentage of internalized CD8-CI-M6PR that reaches the Golgi area was reduced compared to the surrounding non-transfected cells and the non-Golgi internalized CD8-CI-M6PR remained dispersed throughout all the cytoplasm (Fig. 4H).

Overall, our results support that Rab4b is involved in the early endosomes to TGN retrograde trafficking pathway, and that its GTP binding activity, but not its GTPase activity, is required.

### Active Rab4b favors endosomes to TGN retrograde trafficking in a GARP-dependent manner

**Fig. 5:**
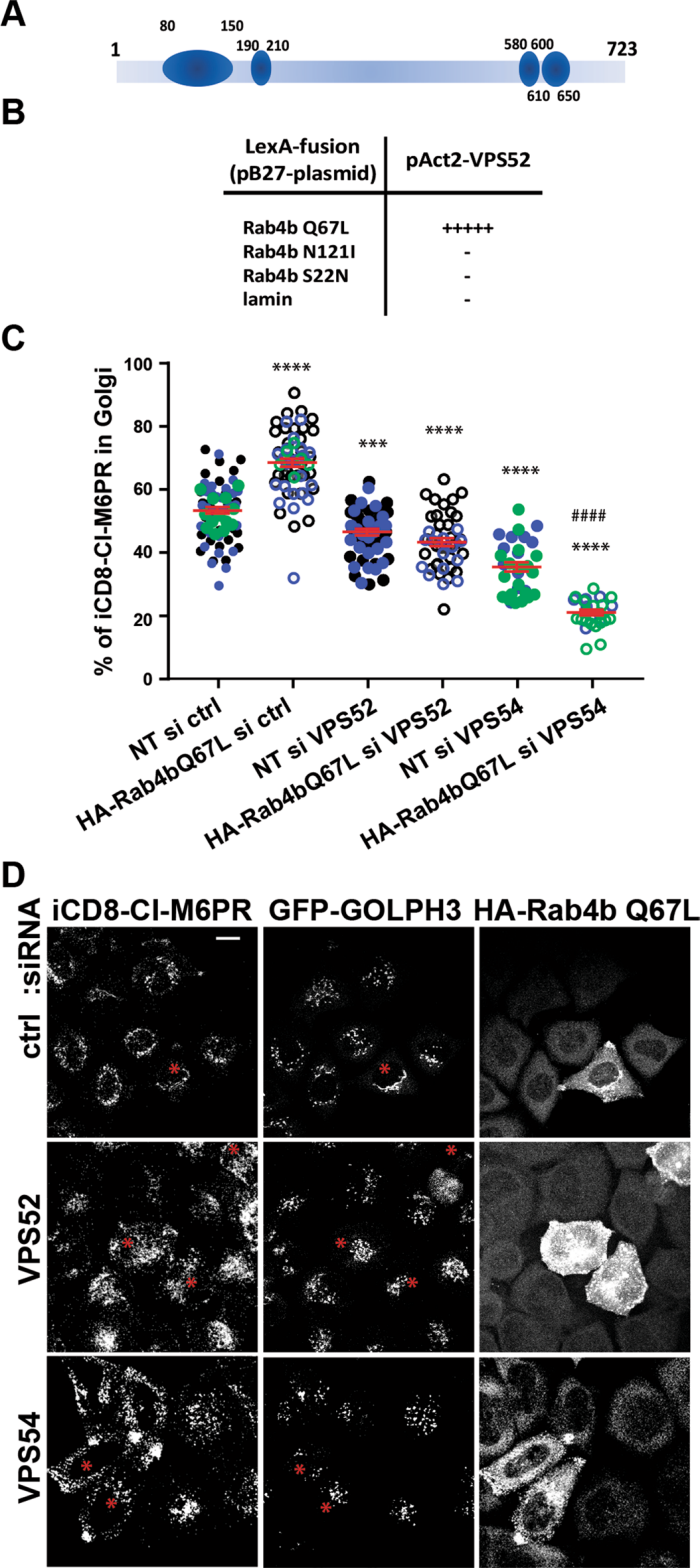
Rab4b-dependent retrograde trafficking involves the GARP complex. **A** Schematic representation of VPS52 structures. The blue circles represent the coiled-coil domain. **B** Schematic representation of the interaction detected in the yeast two-hybrid system, performed as in [26, 27], between full-length VPS52 and the indicated forms of Rab4b or lamin as negative control. + ** +  +  +  + **is for an interaction is detectable before 30 min.—no detectable interaction after 24 h. (**C)** Quantification of iCD8-M6PR in the Golgi area of cells overexpressing HA-Rab4b Q67L (empty circles) and in the surrounding non-transfected cells (NT, filled circles) according to protocol 1 (Additional file 1: Fig. S2A). Each dot represents one cell. Each color (black, blue, or green) indicates independent experiments. ***,**** indicates that the differences are significant compared to NT siCtrl with and ^####^ indicates that the difference is significant compared to NT siVPS54 with p < 0.001 or p < 0.0001 (one-way ANOVA). The significance is the same whether the 3 experiments are analyzed together or independently. **D** Representative images corresponding to the quantification shown in (C), stars are for transfected cells. The downregulation of VPS52 is validated in an independent well in each experiment by Western blot or immunolabeling as shown in (Additional file 1: Fig. S3A-B)

We first confirmed the interaction between VPS52 and Rab4b that was found in the high-throughput screen. We used the active form of Rab4b (Rab4bQ67L) as a bait and the full length VPS52 protein (aminoacid 1 to 723) as the prey in YTH. We found an interaction with Rab4bQ67L, but not with the inactive forms of Rab4b, Rab4bN121I, and Rab4bS22N (Fig. 5A, B), indicating that VPS52 can be an effector of Rab4b.

To explore whether the effect of Rab4b on the retrograde trafficking pathway of CD8-CI-M6PR depended on the GARP complex, we knocked-down VPS52, the effector of Rab4b, or VPS54, the specific subunit of the GARP complex, in cells overexpressing active Rab4b. While we confirmed that the overexpression of active Rab4b increased the targeting of CD8-CI-M6PR to the Golgi area, the efficient downregulation of VPS52 or VPS54 expression (Additional file 1: Fig. S3A) prevented this (Fig. 5C, D). The downregulation of the expression of these components of the GARP complex redistributed the internalized CD8-CI-M6PR to peripheral structures more efficiently in cells overexpressing HA-Rab4b Q67L, where internalized CD8-CI-M6PR and HA-Rab4b Q67L were colocalized, than in the non-transfected surrounding cells (Fig. 5D).

Our results support that the Rab4b-dependent early endosome to TGN retrograde trafficking route requires the GARP complex.

### VPS52 is localized with Rab4b and p230 along the CI-M6PR retrograde trafficking pathway

To strengthen the relationship between VPS52 and Rab4b in the retrograde trafficking route, we examined whether VPS52 is co-localized with GFP-Rab4b and how these two proteins were located relative to the marker of the TGN, p230/golgin-245. VPS52 was present in the Golgi area defined by the presence of p230, but was not particularly enriched there (Additional file 1: Fig. S3C, D). However, we observed some structures containing the three markers (white in the overlay) at the TGN, but also structures containing VPS52 and p230 without GFP-Rab4b (in purple). An orthogonal view of the Golgi area showed that GFP-Rab4b is over the TGN decorated by p230, and when GFP-Rab4b and p230 were in close contact, VPS52 was also present. In the periphery of the cells, there were enlarged vacuoles decorated by GFP-Rab4b and VPS52 without p230. Consistent with this, we found that the co-localization between GFP-Rab4b and VPS52 was greater than between p230 and VPS52 based on both intensity (Additional file 1: Fig. S3C) and object colocalization analyses in 3D reconstruction of confocal sections (Additional file 1: Fig. S3E). In addition, some vesicle-like structures between the Golgi area and the periphery of the cells were only positive for VPS52.

**Fig. 6:**
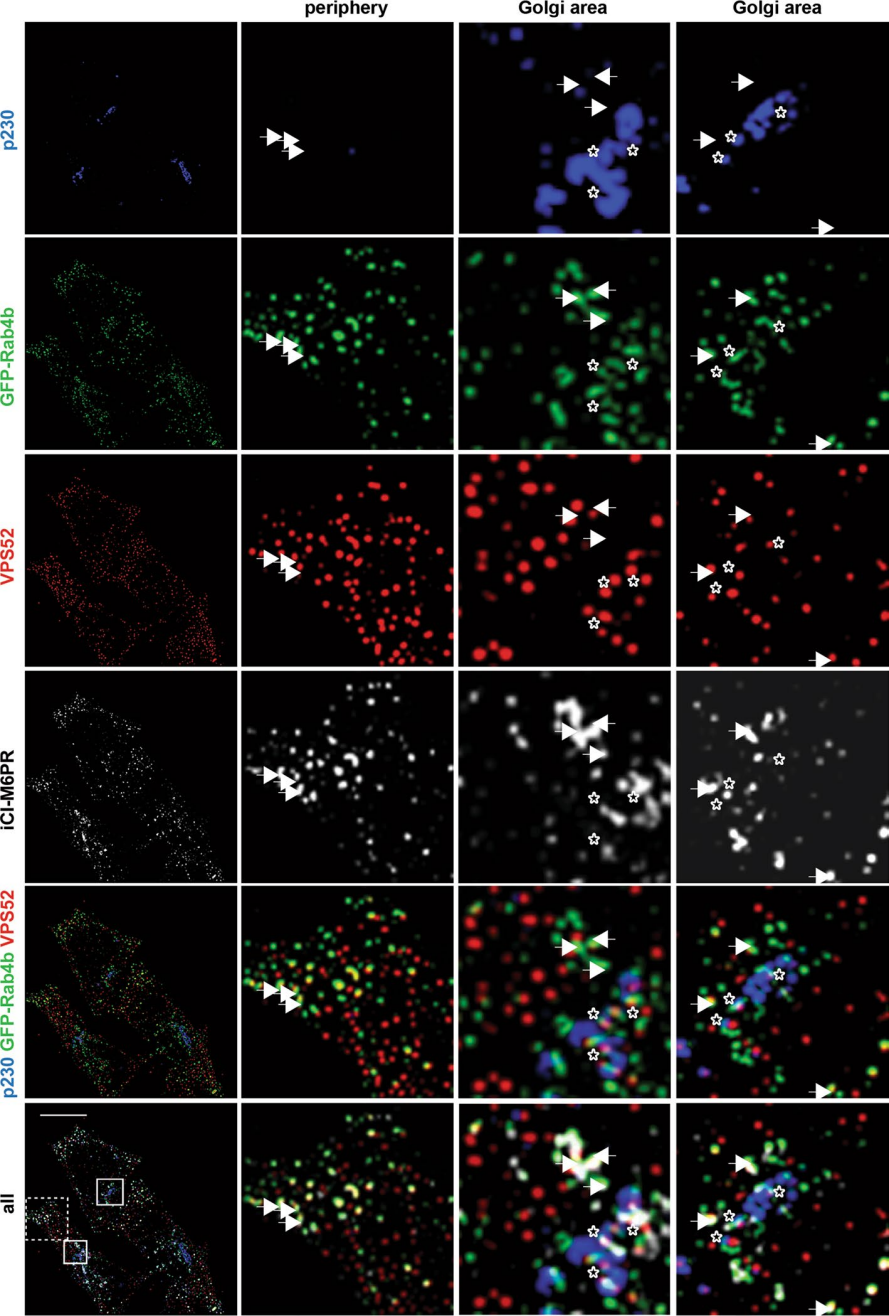
VPS52, Rab4b and p230 localize to the CI-M6PR retrograde pathway. A confocal section taken at the center of cells (left panels) treated with the super-resolution algorithm SRRF. Individual channels were shown with p230 in blue, GFP-Rab4b in green, VPS52 in red, and internalized CI-M6PR (iCI-M6PR) in light grey as well as the merged image of p230, GFP-Rab4b, and VSP52 or of all the channels. Enlarged views of the periphery and Golgi areas are shown, the regions are marked by squares in the whole field of the merge of all colors. Arrows point to vesicles labelled by GFP-Rab4b, VPS52 and iCI-M6PR without p230, whereas the stars point to structures where all the labeling are close together. Bar represents 10 µm

We then determined the distribution of CI-M6PR internalized at steady state between these different compartments. We applied super-resolution radial fluctuation (SRRF) analysis to increase the image resolution (Additional file 1: Fig. S3F). We confirm the extent of colocalization between VPS52, GFP-Rab4, and p230 as a function of the subcellular distribution (Fig. 6). In addition, it was shown that while VPS52 labeling is mostly punctate, GFP-Rab4 decorated tubulo-vesicular structures in contact with VPS52. Internalization of endogenous CI-M6PR (hereafter referred to as internalized CI-M6PR) filled GFP-Rab4b tubulo-vesicular structures in the Golgi area that were in contact with VPS52. This was either separated from the TGN (arrows) or in close contact with the TGN marker p230 (stars). These observations suggest that Rab4b and VPS52 work together to connect Rab4b-positive endosome-derived vesicles to the TGN for the retrograde trafficking of CI-M6PR.

### Rab4b is required for CI-M6PR to traffic from endosome to VPS52-positive vesicle-like structures in route to the TGN

**Fig. 7:**
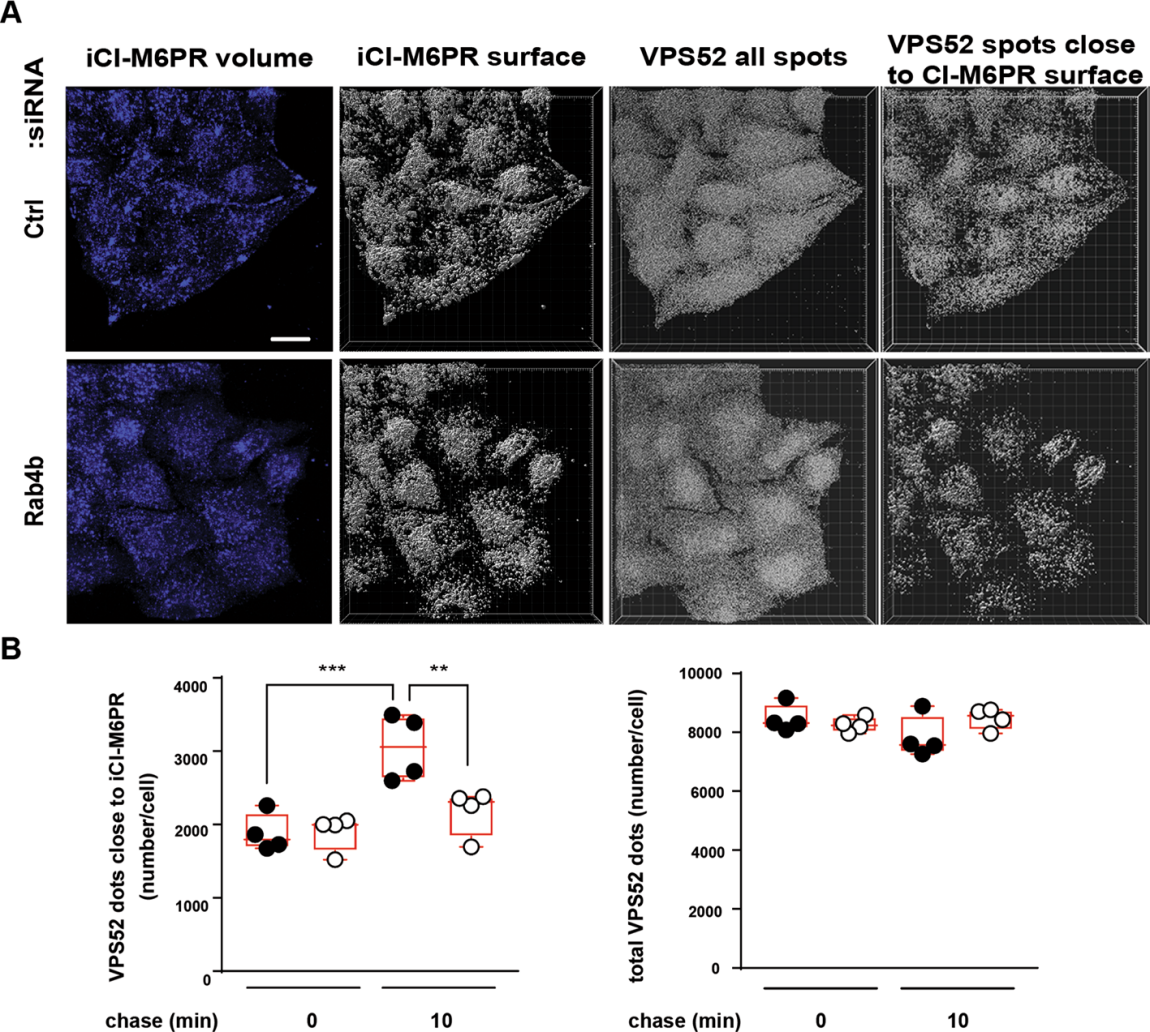
CI-M6PR sorting to endosome carriers is impaired without Rab4b. Internalization of CI-M6PR (iCI-M6PR) is tracked by fluorescently coupled anti-CI-M6PR antibodies using protocol 2 (Additional file 1: Fig. S2A, 10 min chase instead of 30 min) in cells treated with control or anti-Rab4b siRNA. **A** Representative images after the 10 min chase (iCI-M6PR in 3D, after its volume reconstruction, VPS52 converted to spot, and VPS52 spot close to iCI-M6PR volume)The bar is for 20 µm. Image processing is described in the Material and Methods section. The same threshold and the same filters were applied for all the images. **B** Quantification of the number of VPS52 spots near the iCI-M6PR volume in cells treated with control siRNA (filled circles) and anti-Rab4b siRNA (empty circles) before and after the 10 min chase. **, *** indicate that the differences are significant with p < 0.01 and p < 0.001, respectively (one-way ANOVA)

To further elucidated the mechanism by which Rab4b through its effector VPS52 regulated the GARP-dependent retrograde trafficking of CI-M6PR, we knocked-down Rab4b and kinetically followed the retrograde trafficking steps from the endosomes towards the TGN, focusing not only on the cargoes but also on the VPS52-positive structures. First, we observed that the downregulation of Rab4b expression did not alter neither the number of VPS52-positive vesicles, nor the pattern of VPS52 localization within the cells (Fig. 7A, B). Second, using 3D object-based colocalization, as in Additional file 1: Fig. S3, we observed that the arrival of internalized CI-M6PR in VPS52-positive structures was identical in cells expressing or not Rab4b when internalized at 20°C for 15 min. Thirdly, the amount of VPS52-positive structures with internalized CI-M6PR was increased in control cells after 10 min chase at 37°C. However, the downregulation of Rab4b expression reduced the arrival of internalized CI-M6PR in VPS52-positive vesicles (Fig. 7A, B).

**Fig. 8:**
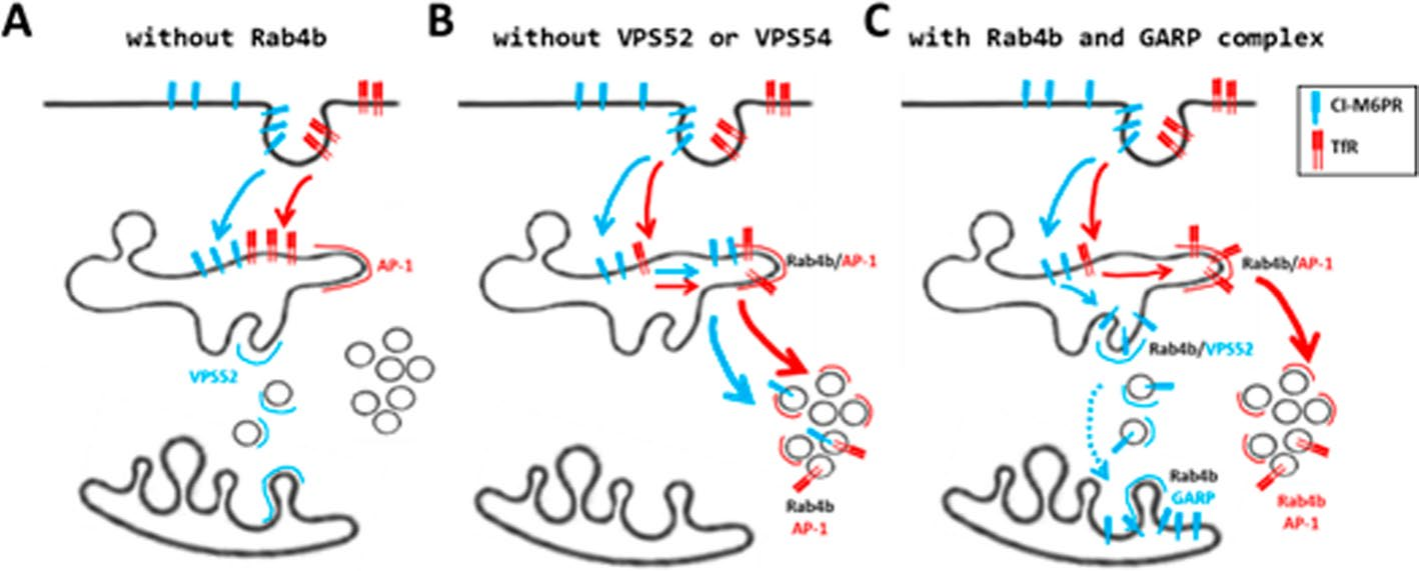
Model for the role of Rab4b in the GARP-dependent retrograde trafficking pathway. The proposed possible model is based on results from this article and previous publications cited in the text. **A** In the absence of Rab4b, CI-M6PR and of the TfR are segregated and their trafficking route to the TGN and the recycling endosomes, respectively, is inhibited. The Rab4b effectors AP-1 and VPS52 remain on the endosomes. **B** In the absence of VPS52 and VPS54, CI-M6PR and of the TfR are not segregated, and they are targeted in a Rab4b/AP-1-dependent

This observation supports a role of Rab4b in the sorting of CI-M6PR from EE to VPS52-positive endosome-derived carriers. This step can therefore be considered as a prerequisite for the acquisition of directional specificity in the sorting of cargo carriers at the early endosomes (Fig. 8).

## Discussion

In the current study, we found that VPS52 interacts with active Rab4b. We designed the experiments to be as close as possible to the biology of the cells to demonstrate that VPS52 requires Rab4b to allow the arrival of the CI-M6PR at the TGN. We thus identified an additional Rab4b-dependent route, distinct from that of the TfR which requires the clathrin adaptor protein AP1 [27]. Furthermore, we showed that in the absence of Rab4b the CI-M6PR is retained in endosomes and cannot efficiently reach VPS52-containing endosome-derived vesicular cargo carriers.

The arguments that allow us to propose that Rab4b acts in the VPS52-dependent retrograde trafficking route of the CI-M6PR are as follows. We have shown that the overexpression of an active form of Rab4b increases the arrival of CI-M6PR at the TGN, whereas the overexpression of inactive Rab4b and the downregulation of its expression have the opposite effect. In addition, Rab4b is partially localized at the TGN and the downregulation of Rab4b expression induces partial misrouting of CI-M6PR to lysosomes, accompanied by its degradation, a consequence already described when defects in the endosomes-to-TGN retrograde trafficking pathway were experimentally induced [32–34]. More importantly, we found that the downregulation of VPS52 impaired the ability of active Rab4b to enhance the trafficking of CI-M6PR to the TGN. We believe that the GARP complex, rather than the EARP complex, is involved in the new trafficking route of CI-M6PR that we have just described. Indeed, we found that the downregulation of VPS54, the unique subunit of the GARP complex, also disrupts the effect of Rab4b overexpression on the CI-M6PR trafficking to the TGN.

Although CI-M6PR can take different routes to reach the TGN, i.e. directly from the early endosomes, via the recycling endosomes, or via late endosomes/lysosomes [35–37] [38, 39], we favor a direct early endosomal pathway to the TGN. Indeed, we found that Rab4b is required for the segregation of TfR, the archetypal cargo for early endosomes to recycling endosomes trafficking, and CI-M6PR. However, we cannot exclude the possibility that CI-M6PR retrograde trafficking uses an intermediate compartment to reach the TGN. Indeed, internalized CI-M6PR accumulates in a peripheral compartment when VPS54 expression is decreased. Rab4b would therefore be able to control two successive steps of CI-M6PR trafficking, the first being GARP-independent from early endosomes to the peripheral intermediate compartment, and the second being GARP-dependent from this compartment to the TGN. Alternatively, this could be a default pathway that is rarely present in control cells, due to the lack of CI-M6PR and TfR segregation at the early endosomes. If so, this implies that Rab4b controls two sorting events at the endosomes, that of TfR, which requires an interaction between Rab4b and AP1 [27], and that of CI-M6PR, which requires the GARP complex. Notably, this is not the first time that such a situation has been reported [40–42]. A same Rab proteins can additionally be involved both in the control of intracellular trafficking and in other unrelated functions involving their ability to organize membrane subdomains, such as mitophagy [43, 44], the organization of signaling platforms [45], the organization of SNARE complexes, or more recently the localization of a specific subset of mRNA on endosomes [46]. Understanding how a same Rab protein can control all of its assigned functions will be a major challenge in cell biology.

Because the GARP complex intervenes to prepare for fusion when the endosome-derived retrograde carriers and the tubulo-vesicular TGN are close enough [12], we expected to find a clear enrichment of VPS52 at the TGN, which is not the case. Rather, our results support that endogenous VPS52 is more likely to localize to endosome-derived structures, which is not the case for the overexpressed VPS52 and the other overexpressed GARP subunits, all of which are enriched at the TGN [47]. Importantly, VPS52 co-localizes with Rab4b along the entire retrograde trafficking pathway from early endosomes to the TGN of the CI-M6PR and the downregulation of Rab4b expression limits the arrival of internalized CI-M6PR into VPS52-positive endosomal microdomains from which endosome-derived carriers decorated by VPS52 could be formed.

Rab4b on the endosomal side and Arl5 at the TGN will allow the GARP complex to bridge the two compartments when they are close enough. This is similar to what has recently been described for the HOPS multisubunit complex, which bridges late endosomes to the lysosomes by interacting with late endosomal Rab2 and lysosomal Arl8 [48]. However, Rab2 has not been shown to be involved in sorting receptors/transporters into HOPS-containing domains of the late endosomes.

An open question is whether the entire GARP complex is present on these microdomains and on the endosome-derived carriers. Indeed, the overexpressed VPS54 subunit is exclusively localized to the TGN and no data are available on the endogenous localization of VPS54 subunits. As it has recently been shown that VPS52 is present on the endosomes apart from the GARP tethering complex [49], Rab4b could alternatively be important to allow the formation of the full functional GARP complex by allowing its reassembly. Of note, the observation that the subunits of the same complex do not have a completely overlapping distribution has already been described [50], suggesting that tethering complexes are not necessary preformed at the fusion site.

## Conclusion

Taken together, our observations may support the concept that Rab4b coordinates the molecular mechanisms involved in the sorting of CI-M6PR towards VPS52-positive endosomal nanodomains, thereby conferring a directional specificity to the retrograde trafficking pathway. This initial step of the retrograde trafficking is critical for the GARP-dependent tethering of endosome-derived vesicular carriers at the TGN and their subsequent fusion. The retrograde trafficking route is important for nutrient and divalent metal ion homeostasis, lysosomal functions, and activation of signaling pathways. Dysregulation in this trafficking route has been implicated in metabolic, neurological, and immune diseases. Our discovery provides additional molecular mechanisms to be considered in pathological situations where the retrograde trafficking is impaired without yet knowing the molecules involved.

### Supplementary Information

**Additional file 1: Figure S1.** (related to Figure 1). **A** Index of co-localization between Rab4b and organelle markers. Colocalization between organelle markers and similar level of overexpressed GFP-Rab4b. **B** Analysis of the co-localization between EEA1 and GFP-Rab4b wt or inactive. Object-based quantification of the number of structures positive for the indicated proteins. **Figure S2.** (related to Fig. 4). The down-modulation of Rab4b inhibits retrograde trafficking. **A** Design used to study the endosome to Golgi route. **B** Images quantified in Fig. 4E-F. **C** Confocal images iTf, iChTx, and GM130 with control or anti-Rab4b siRNA. **D** Same as Fig. 4G, with anti-VPS54 siRNA. **Figure S3.** (related to Fig. 5-6). **A** VPS52 immunoblot. **B** VPS52 labelling with control or anti-VPS52 siRNA. **C** 3D-reconstruction of Z-stacks. Intensity-based colocalization (white). **D** Z-orthoslice **E** 3D object-based colocalization; 3D-surface rendering (of GFP-Rab4b/p230), dots for (VPS5. **F** Confocal images (Fig. 6) before and after SSRF algorithm.

## Data Availability

All the materials produced in this study are available upon request. Data availability: Not applicable.

## Abbreviations

ChTx: Cholera toxin
GARP: Golgi-associated retrograde protein
iTf: Internalized transferrin
iCI-M6PR: Internalized mannose-6-phosphate receptor
SRRF: Super-resolution Radial Fluctuation
TEM: Transmission Electron Microscoy
TGN: *trans* Golgi Network
TfR: Transferrin receptor
WT: Wild type
