# Supplementary material for: Golgi-associated retrograde protein (GARP) complex-dependent endosomes to trans Golgi network retrograde trafficking is controlled by Rab4b
Source: Cell Mol Biol Lett. 2024 Apr 16;29:54. doi: 10.1186/s11658-024-00574-w (PMC11020649; doi:10.1186/s11658-024-00574-w)
Supplement: Supplementary file 1 — Additional file 1: Figure S1. (related to Figure 1). A Index of co-localization between Rab4b and organelle markers. Colocalization between organelle markers and similar level of overexpressed GFP-Rab4b. B Analysis of the co-localization between EEA1 and GFP-Rab4b wt or inactive. Object-based quantification of the number of structures positive for the indicated proteins. Figure S2. (related to Fig. 4). The down-modulation of Rab4b inhibits retrograde trafficking. A Design used to study the endosome to Golgi route. B Images quantified in Fig. 4E-F. C Confocal images iTf, iChTx, and GM130 with control or anti-Rab4b siRNA. D Same as Fig. 4G, with anti-VPS54 siRNA. Figure S3. (related to Fig. 5-6). A VPS52 immunoblot. B VPS52 labelling with control or anti-VPS52 siRNA. C 3D-reconstruction of Z-stacks. Intensity-based colocalization (white). D Z-orthoslice E 3D object-based colocalization; 3D-surface rendering (of GFP-Rab4b/p230), dots for (VPS5. F Confocal images (Fig. 6) before and after SSRF algorithm. [file 11658_2024_574_MOESM1_ESM.pdf]

**Additional file 1**

# Golgi-associated retrograde protein (GARP) complex-dependent endosomes to trans Golgi network retrograde trafficking is controlled by Rab4b

**Gilleron *et al***

**Supplemental Figure 1 (related to Figure 1). (A) Index of co-localization between Rab4b and organelle markers.** Colocalization between organelle markers and similar level of overexpressed GFP-Rab4b. (**B**) **Analysis of the co-localization between EEA1 and GFP-Rab4b wt or inactive.** Object-based quantification of the number of structures positive for the indicated proteins.

**Supplemental Figure 2 (related to Fig. 4). The down-modulation of Rab4b inhibits retrograde trafficking.** (**A**) Design used to study the endosome to Golgi route. (**B**) Images quantified in Fig. 4E-F. (**C**) Confocal images iTf, iChTx, and GM130 with control or anti-Rab4b siRNA. (**D**). Same as Fig. 4G, with anti-VPS54 siRNA.

**Supplemental Figure 3 (related to Fig. 5-6).** **(A)** VPS52 immunoblot. **(B)** VPS52 labelling with control or anti-VPS52 siRNA. **(C)** 3D-reconstruction of Z-stacks. Intensity-based colocalization ( white). **(D)** Z-orthoslice **(E)** 3D object-based colocalization; 3D-surface rendering (of GFP-Rab4b /p230), dots for (VPS5. (F) Confocal images (Fig. 6) before and after SSRF algorithm.

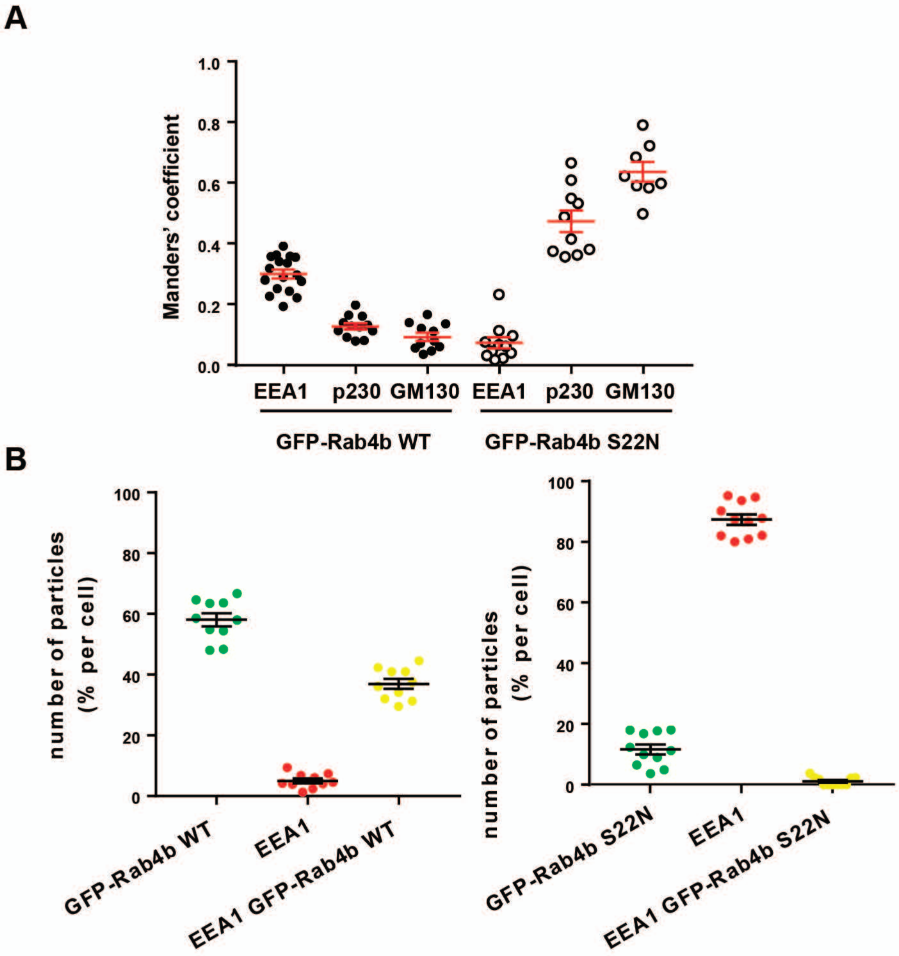


# Supplemental Figure 1

*(related to Figure 1)*

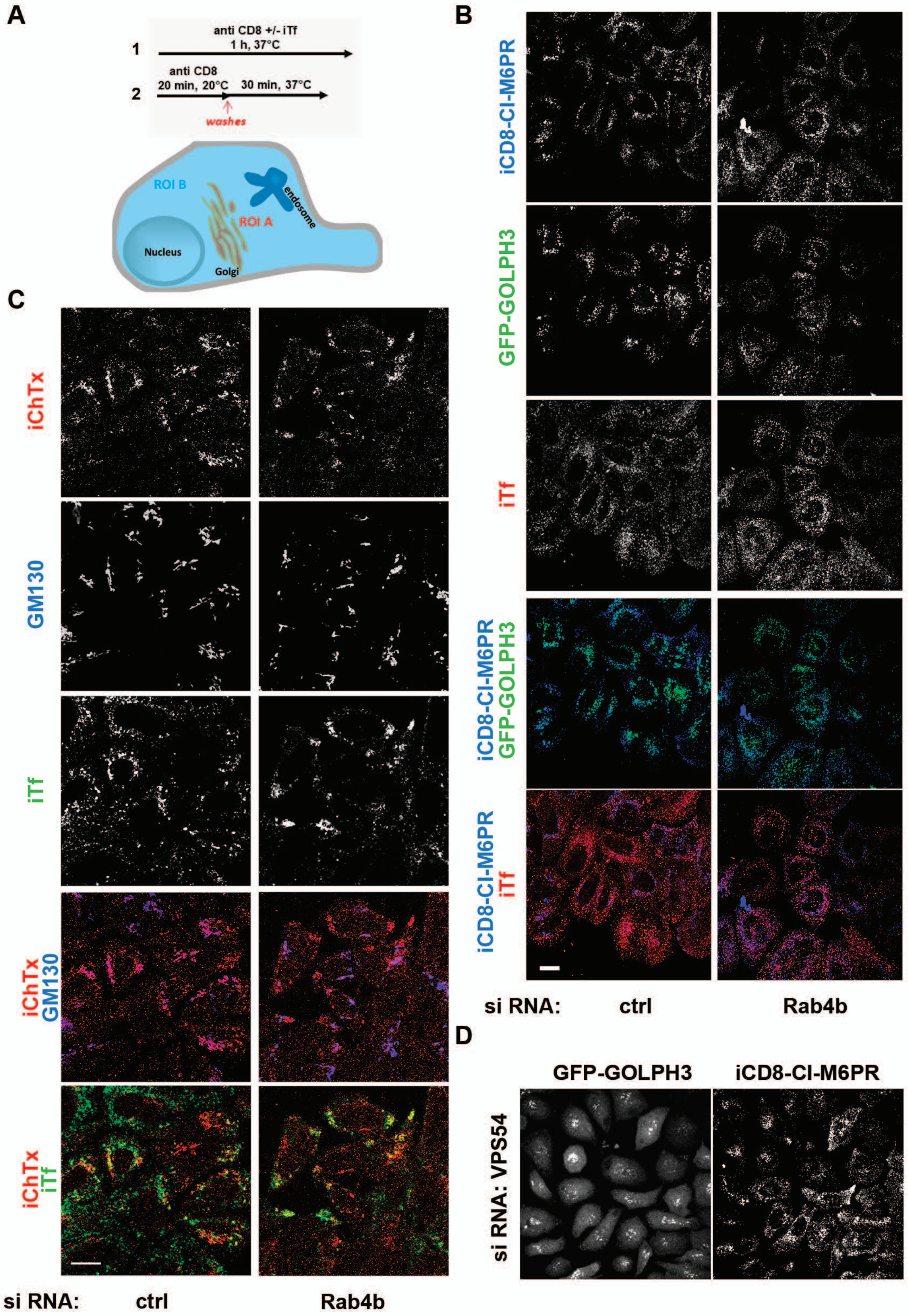


# Supplemental Figure 2

*(related to Figure 3-4)*

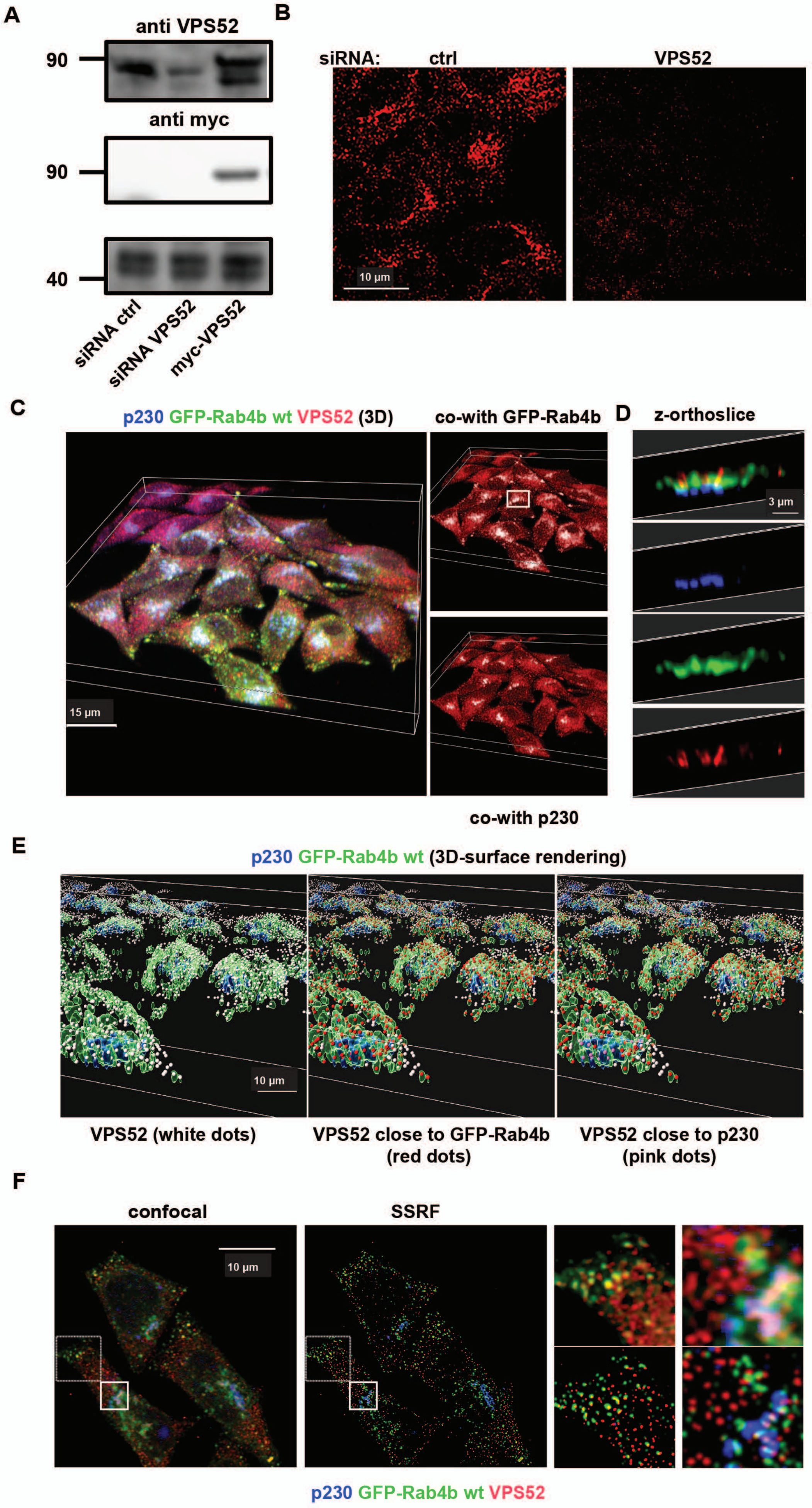


# Suppl Figure 3_Fig5-6
